# Impact of large volume paracentesis on respiratory parameters including transpulmonary pressure and on transpulmonary thermodilution derived hemodynamics: A prospective study

**DOI:** 10.1371/journal.pone.0193654

**Published:** 2018-03-14

**Authors:** Ulrich Mayr, Eugen Karsten, Tobias Lahmer, Sebastian Rasch, Philipp Thies, Benedikt Henschel, Gerrit Fischer, Roland M. Schmid, Wolfgang Huber

**Affiliations:** Klinik und Poliklinik für Innere Medizin II, Klinikum rechts der Isar, Technische Universität München, Ismaninger Strasse 22, München, Germany; Duke University, UNITED STATES

## Abstract

**Introduction:**

Appropriate mechanical ventilation and prevention of alveolar collaps is mainly dependent on transpulmonary pressure TPP. TPP is assessed by measurement of esophageal pressure EP, largely influenced by pleural and intraabdominal pressure IAP. Consecutively, TPP-guided ventilation might be particularly useful in patients with high IAP.

This study investigates the impact of large volume paracentesis LVP on TPP, EP, IAP as well as on hemodynamic and respiratory function in patients with liver cirrhosis and tense ascites.

**Material and methods:**

We analysed 23 LVP-procedures in 11 cirrhotic patients ventilated with the AVEA Viasys respirator (CareFusion, USA) which is capable to measure EP via an esophageal tube.

**Results:**

LVP of a mean volume of 4826±1276 mL of ascites resulted in marked increases in inspiratory (17.9±8.9 vs. 5.4±13.3 cmH_2_O; p<0.001) as well as expiratory TPP (-3.0±4.7 vs. -15.9±10.9 cmH_2_O; p<0.001; primary endpoint). In parallel, the inspiratory (2.4±8.7 vs. 14.1±14.5 cmH_2_O; p<0.001) and expiratory EP (12.4±6.0 vs. 24.9±11.3 cmH_2_O; p<0.001) significantly decreased. The effects were most pronounced for the release of the first 500 mL of ascites. LVP evoked substantial decreases in IAP and central venous pressure CVP. By contrast, mean arterial pressure, cardiac index, global end-diastolic volume index, extravascular lung water index and systemic vascular resistance index did not change.

Among the respiratory parameters we observed an increase in p_a_O_2_/F_i_O_2_ (247.7±60.9 vs. 208.3±46.8 mmHg; p<0.001) and a decrease in Oxygenation Index OI (4.8±2.0 vs. 5.8±3.1 cmH_2_O/mmHg; p = 0.002). Tidal volume (510±100 vs. 452±113 mL; p = 0.008) and dynamic respiratory system compliance C_dyn_ (46.8±15.9 vs. 35.1±14.6 mL/cmH_2_0; p<0.001) increased, whereas p_a_CO_2_ (47.3±10.7 vs. 51.2±12.3mmHg; p = 0.046) and the respiratory rate decreased (17.1±7.3 vs. 19.6±7.8 min^-1^; p = 0.010).

**Conclusions:**

In mechanically ventilated patients with decompensated cirrhosis, intraabdominal hypertension resulted in a substantially decreased TPP despite PEEP-setting according to the ARDSNet.

In these patients LVP markedly increased TPP and improved respiratory function in parallel with a decline of EP. Furthermore, LVP induced a decrease in IAP and CVP, while other hemodynamic parameters did not change.

## Introduction

Mechanical ventilation (MV) in acute respiratory distress syndrome (ARDS) is guided by a number of standard recommendations for key parameters such as positive end-expiratory pressure (PEEP) and tidal volume (TV) [1, 2]. In addition to these general recommendations modern strategies try to optimize the ventilator setting for the individual patient. Due to complex interactions of MV with extrapulmonary organ functions, personalized ventilator setting should also consider the underlying disease. To account for the individual pulmonary (patho)physiology, modern respirators have become diagnostic tools in addition to their therapeutic purpose. An increasing number of volumes, pressures and flows are routinely and continuously provided by advanced respirators [3, 4]. Until recently this ventilator-monitoring was mainly based on the measurement of *intrapulmonary* airway pressures and volumes. However, sufficient ventilation depends not only on *intrapulmonary* pressures, but to a great extent on the *extrapulmonary* pressure level. The latter largely varies in critically ill patients, and it is highly dependent on both pleural and intraabdominal pressures [5, 6]. “*Extrapulmonary*” or pleural pressure usually is estimated by measurement of the esophageal pressure (EP) using esophageal catheters [7, 8] which allows monitoring of transpulmonary pressure (TPP). TPP is defined as the difference between the airway pressure (PAW) and EP. TPP is crucial for lung-distending and overcoming chest wall elastance [9]. Consequently, modern ventilator strategies aim at optimization of TPP to avoid recurring alveolar collapse as well as over-distension [10]. In patients with ARDS, a strategy adjusting PEEP according to TPP improved outcome compared to current guideline-based treatment [11].

Ventilator setting in patients with liver cirrhosis is particularly challenging due to several reasons: Previous studies demonstrated that the need for mechanical ventilation is independently associated with the ICU mortality of patients with decompensated liver cirrhosis [12–14]. The accumulation of ascites is a typical complication of end-stage liver disease. Ascites impairs lung function due to an increase of intraabdominal pressure (IAP) [6, 15–17].

Therefore, TPP-guided ventilator setting might be of particular importance in patients with tense ascites and increased IAP. A number of studies in ventilated patients with cirrhosis suggest that large volume paracentesis LVP [18–20] improves lung compliance and oxygenation [21–24]. Furthermore, one of these studies using trans-pulmonary thermodilution (TPTD) technique demonstrated hemodynamic stability in addition to markedly improved respiratory function [25].

Despite their unquestioned merits none of these studies investigated the impact of LVP on TPP. Therefore, our study aimed to investigate the effect of LVP on inspiratory and expiratory TPP (primary endpoint), IAP and hemodynamics in patients with decompensated liver cirrhosis equipped with monitoring of EP as well as with TPTD.

## Materials and methods

### Study design

This prospective observational study was approved by the institutional review board (Ethikkommission Technische Universität München; Fakultät für Medizin; Project number 5384/12). Informed consent was obtained by all patients or their legal representatives.

Between January 2012 and July 2014 all patients of an eight-bed university hospital general ICU with decompensated liver cirrhosis under mechanical ventilation were screened for the feasibility of EP measurements, if LVP had to be performed irrespective of the study and based on the indication made by the treating ICU physician. EP-analysis was considered to be feasible in patients without high-grade esophageal varices (grad III or IV and/or risk signs like cherry red spots or “varix on varix phenomenon”) and without a history of gastrointestinal bleeding within last 6 months.

Finally, a total of 23 LVP-procedures in 11 patients with decompensated cirrhosis and tense ascites were analyzed.

### Techniques

#### Ventilation

In general, eligible patients were transferred from the routine ventilator device EVITA XL of our ICU (Dräger, Lübeck, Germany) to the AVEA Viasys ventilator (CareFusion, San Diego; USA). Key ventilation parameters were set according to ARDSNet recommendations for PEEP and low tidal volume [1, 2]. This ventilator setting—based on medical assessment by the treating ICU physician irrespective of the study—was not changed after the transfer to the AVEA device and initiation of EP measurement with an AVEA esophageal tube (SmartCath Nasogastric Pressure Monitoring Tube Set, 16 FR). This tube provides EP measurement via an air-inflatable ballon and can be placed identically to a conventional nasogastric tube, hereby also allowing for enteral nutrition. The AVEA ventilator continuously analyzed levels of airway pressure PAW, EP and the resulting TPP.

Routine parameters of ventilator setting such as PEEP, tidal volume TV, mean airway pressure, dynamic respiratory system compliance C_dyn_ and fraction of inspired oxygen F_i_O_2_ were recorded at baseline, during and at the end of paracentesis. P_a_O_2_ and p_a_CO_2_ were measured using a fully-automatic blood gas analysis device (Rapid Point 400, Siemens Healthcare Diagnostic GmbH, Eschborn, Germany). Blood gas analysis and ventilatory parameters were used for calculation of P_a_O_2_/F_i_O_2_ as well as Oxygenation Index (OI = F_i_O_2_*mean airway pressure*100/P_a_O_2_) [26].

#### LVP and measurement of IAP

LVP was performed in supine position and guided by ultrasound [27]. Two techniques of IAP-monitoring were used in parallel: IAP was determined by *intra-peritoneal* measurement (IAP_P) connecting the puncture needle with a pressure transducer and in addition by measurement of the *intra-vesical* (IAP-V) pressure [28, 29]. Substitution of albumin was performed after LVP according to the current guidelines [30].

#### Hemodynamic monitoring

All patients were under hemodynamic monitoring with the PiCCO-2-device (Pulsion Medical Systems SE, Feldkirchen, Germany) irrespective of the study. Hemodynamic monitoring using TPTD was performed as previously described [31, 32]: A 5 Fr thermistor-tipped arterial line (Pulsiocath, Pulsion Medical Systems SE, Feldkirchen Germany) inserted through a femoral artery and a hemodynamic monitor (PiCCO-2, Pulsion Medical Systems SE, Feldkirchen Germany) were used to derive and analyze the thermodilution curve after injection of a cold indicator bolus (15ml of saline) through a central venous catheter. Measurements were done in triplicate, averaged and automatically indexed according to the manufacturer´s recommendation. Central venous pressure (CVP) was measured via the central venous catheter at end-expiration.

### Data collection

Clinical and laboratory parameters for the calculation of APACHE II-, SOFA-, MELD- and Child-Pugh-scores were recorded on the day of paracentesis. Ventilatory parameters including TPP and EP as well as IAP were documented immediately before as well as after the release of 500, 1000, 1500, 3000, 4500 and up to a maximum volume of 6000 mL ascites. Parameters of hemodynamic and respiratory function were documented immediately before and after paracentesis.

### Statistical analysis, power calculation

#### Primary endpoint and statistics

For primary outcome analysis we investigated expiratory TPP at the end of LVP compared to baseline. Assuming a decrease in at least 80% of 20 LVPs resulted in a statistical power of at least 80% with a p-value < 0.05 (two-tail-test).

All analyses were performed using IPM SPSS Statistics 23 (IMB Corp.; Armonk; NY); graphs were generated using GraphPad Prism 6.0 (GraphPad Software, La Jolla, CA, USA). Correlations were calculated using Spearman`s correlation coefficient. Continuous variables are expressed as mean±standard deviation. Categorical variables are expressed as percentages. To compare continuous variables we used Wilcoxon-test for paired samples. Significance was assumed at a p-value < 0.05.

## Results

### Patients´ characteristics

Patients´ baseline characteristics are presented in Table 1.

**Table 1:** Patients´ baseline characteristics.

Patients´ baseline characteristics	
**Male sex, n/total (%)**	5/11 (45%)
**Age, years**	51±15
**Body weight, kg**	77±21
**Body height, cm**	173±9
**APACHE II**	24±8 (13–36)
**SOFA**	14±4 (7–20)
**MELD**	27±10(19–40)
**Child-Pugh**	10.4±2.4 (8–13)
**Child C, n/total (%)**	10/11 (91%)
**Etiology of cirrhosis, n/total (%)**	Alcoholic 7/11 (64%)
	Viral 2/11 (18%)
	Cryptogenic 2/11 (18%)
**Mode of ventilation, n/total (%)**	Pressure-controlled 3/11 (27%)
	Pressure-supported 8/11 (73%)
**Indication for ventilation, n/total (%)**	Pneumonia, Sepsis 8/11 (73%)
	Hepatic encephalopathy 3/11 (27%)
**Baseline PEEP-level, cmH**_**2**_**O**	7.7±2.0 (6–14)
**Baseline F**_**i**_**O**_**2**_**, %**	42.0±1.1 (30–70)

APACHE: Acute physiology and chronic health evaluation

SOFA: Sequential organ failure assessment

MELD: Model of end-stage liver disease

PEEP: Positive end-expiratory pressure

F_i_O_2_: Fraction of inspired oxygen

23 LVP-procedures in 11 patients (6 female, 5 male) with decompensated liver cirrhosis and tense ascites were performed. The etiology of cirrhosis was predominantly alcoholic. APACHE, SOFA, MELD and Child-Pugh scores were in line with critical illness and advanced hepatic impairment. Patients were mechanically ventilated due to respiratory insufficiency (n = 8) or hepatic encephalopathy (n = 3). Ventilation was performed as pressure-controlled (n = 3 patients; 7 datasets) or pressure-supported ventilation (n = 8 patients; 16 datasets). Mean PEEP-level was 7.7±2.0 (6–14) cmH_2_0 and mean F_i_O_2_ 42.0±11.1 (30–70) %. Both parameters were maintained constant during LVP.

For all patients the PEEP was set according to the ARDSNet recommendations for the individual F_i_O_2_ according to the local standard: As shown in Table 1, minimum, mean and maximum values of PEEP and F_i_O_2_ were in line with combinations of both parameters suggested by the ARDSNet.

### Large volume paracentesis, transpulmonary, esophageal and intraabdominal pressures

**Fig 1:**
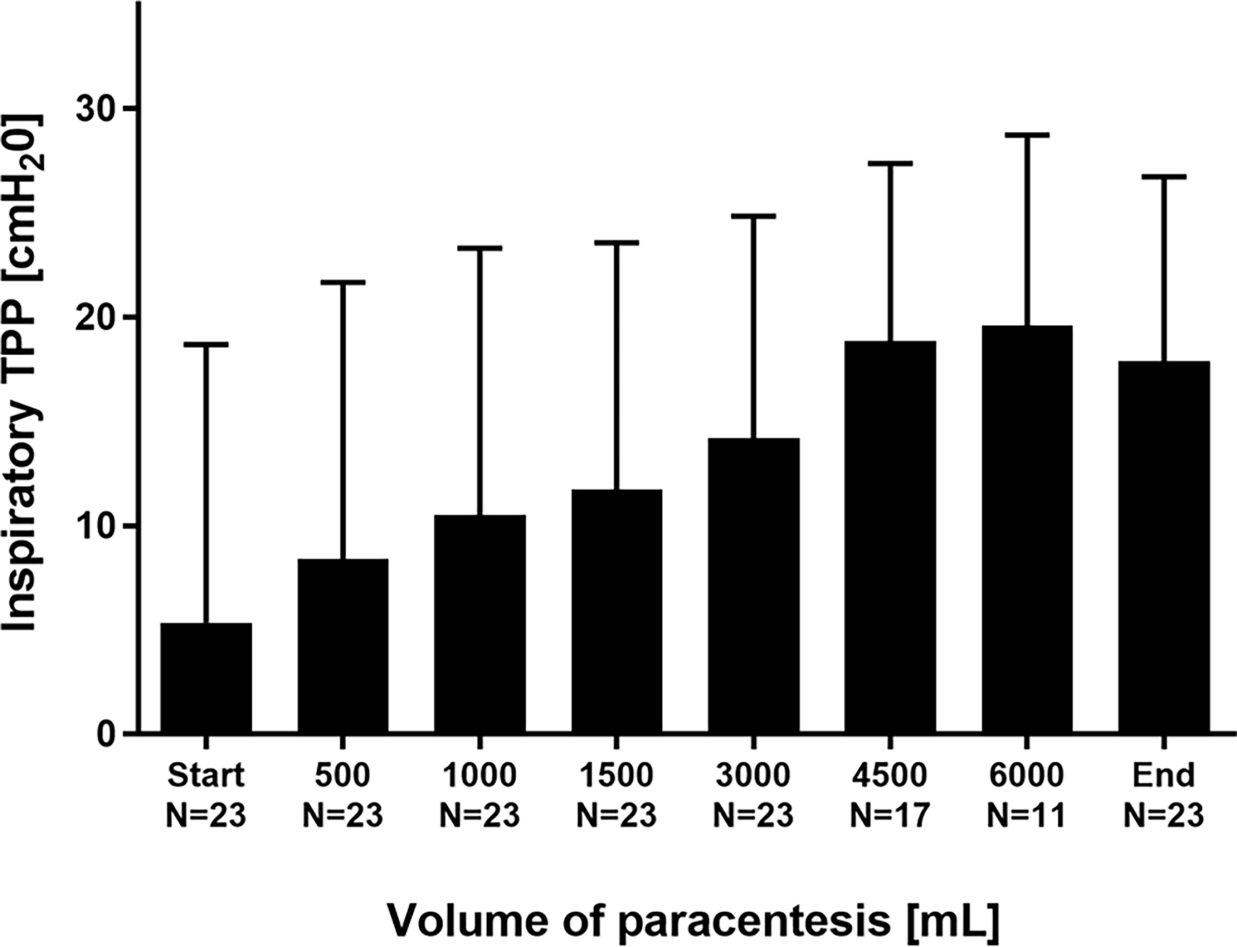
Inspiratory transpulmonary pressure TPP in the course of stepwise release of ascites.

**Fig 2:**
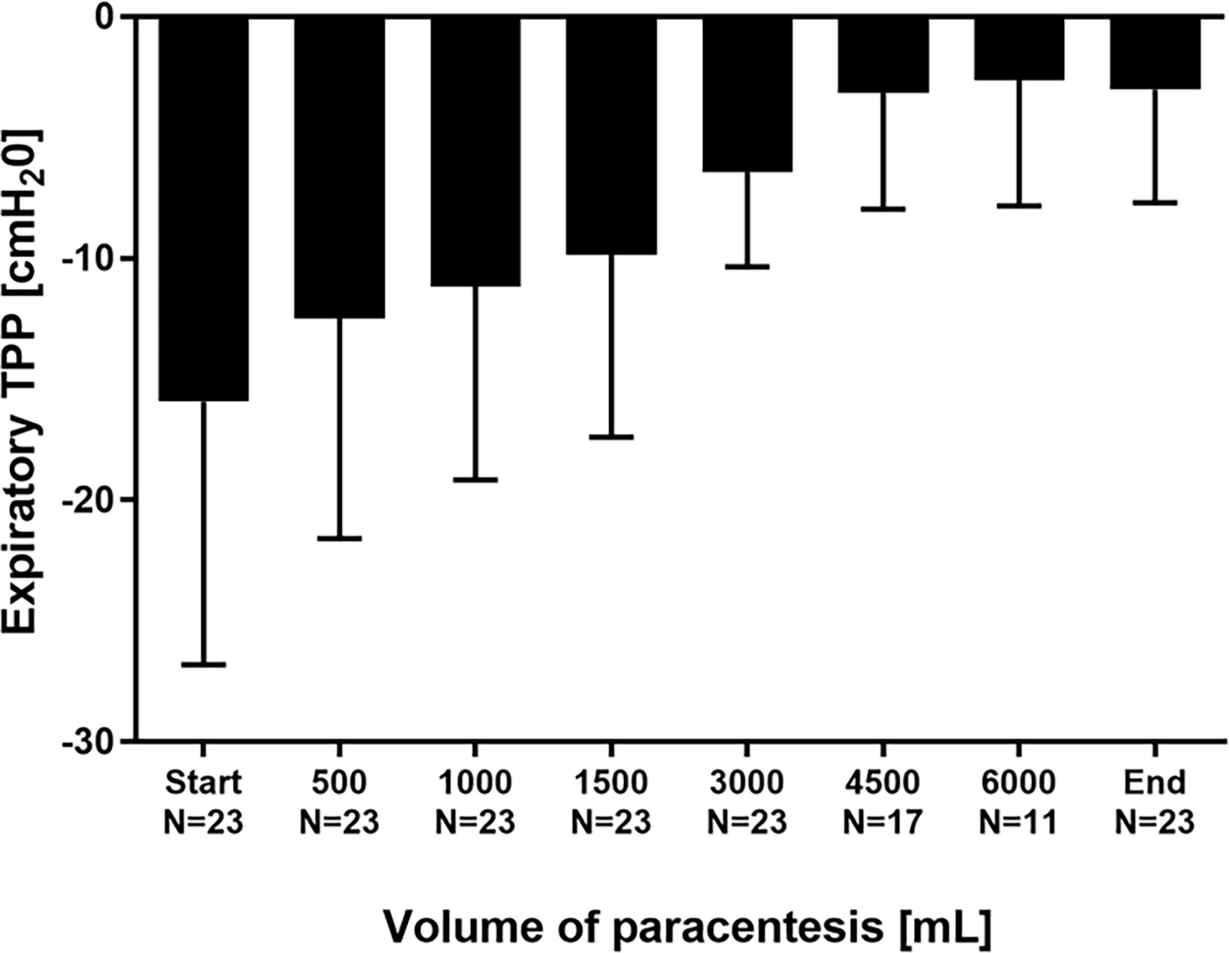
Expiratory transpulmonary pressure TPP in the course of stepwise release of ascites.

23 LVP-procedures with a mean volume of 4826±1276 mL removed ascites (≥3000 mL in all 23 LVPs, 4500 mL in 17 LVPs, 6000 mL in 11 LVPs) were analyzed. LVP resulted in a marked increase of mean TPP: At the end of LVP, inspiratory TPP increased from 5.4±13.3 to 17.9±8.9 cmH_2_O (p<0.001; Fig 1). Expiratory TPP increased from -15.9±10.9 to -3.0±4.7 cmH_2_0 (p<0.001; Fig 2). Despite these increases, mean expiratory TPP remained in the negative range, when patients were ventilated with the preset PEEP-level according to the ARDSNet recommendations.

**Fig 3:**
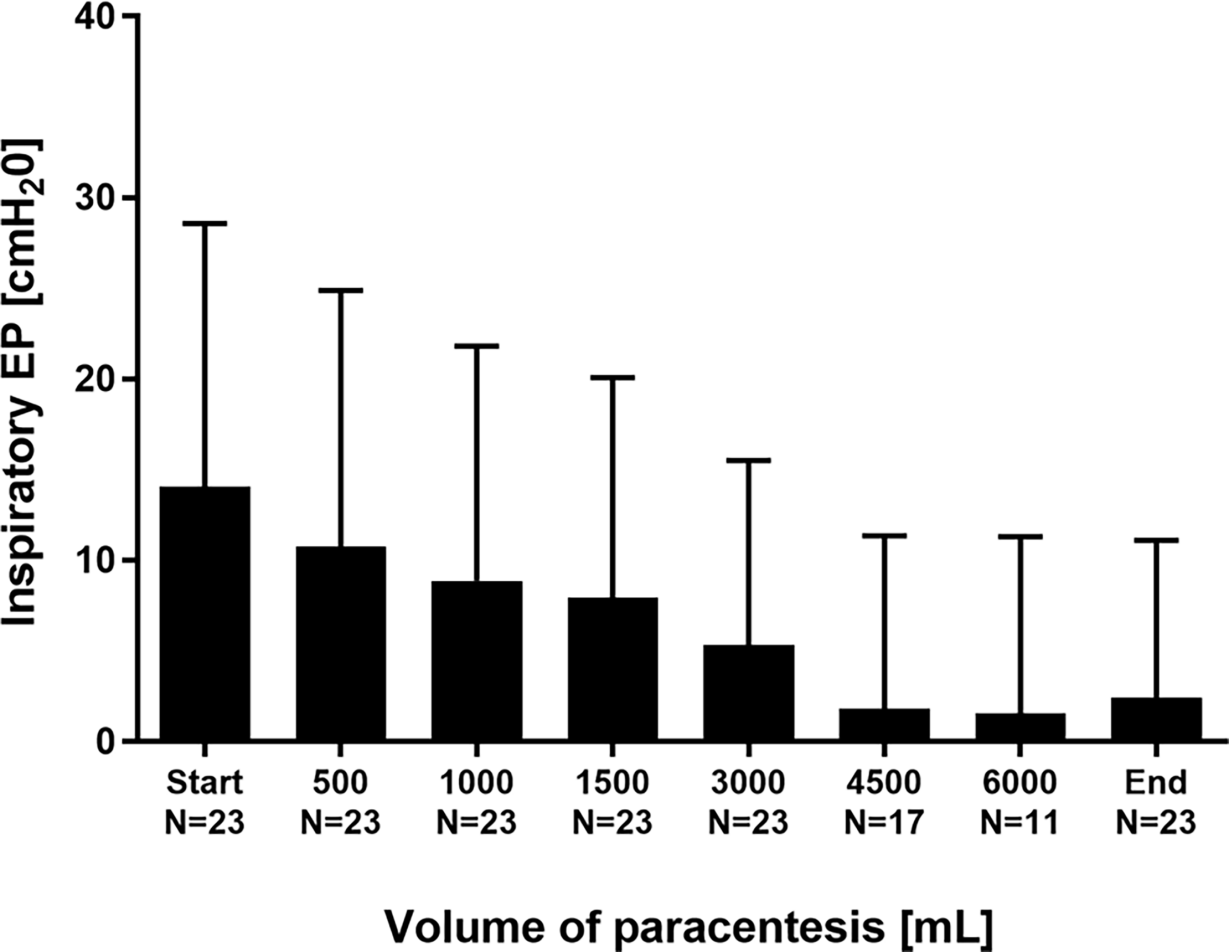
Inspiratory esophageal pressure EP in the course of stepwise release of ascites.

**Fig 4:**
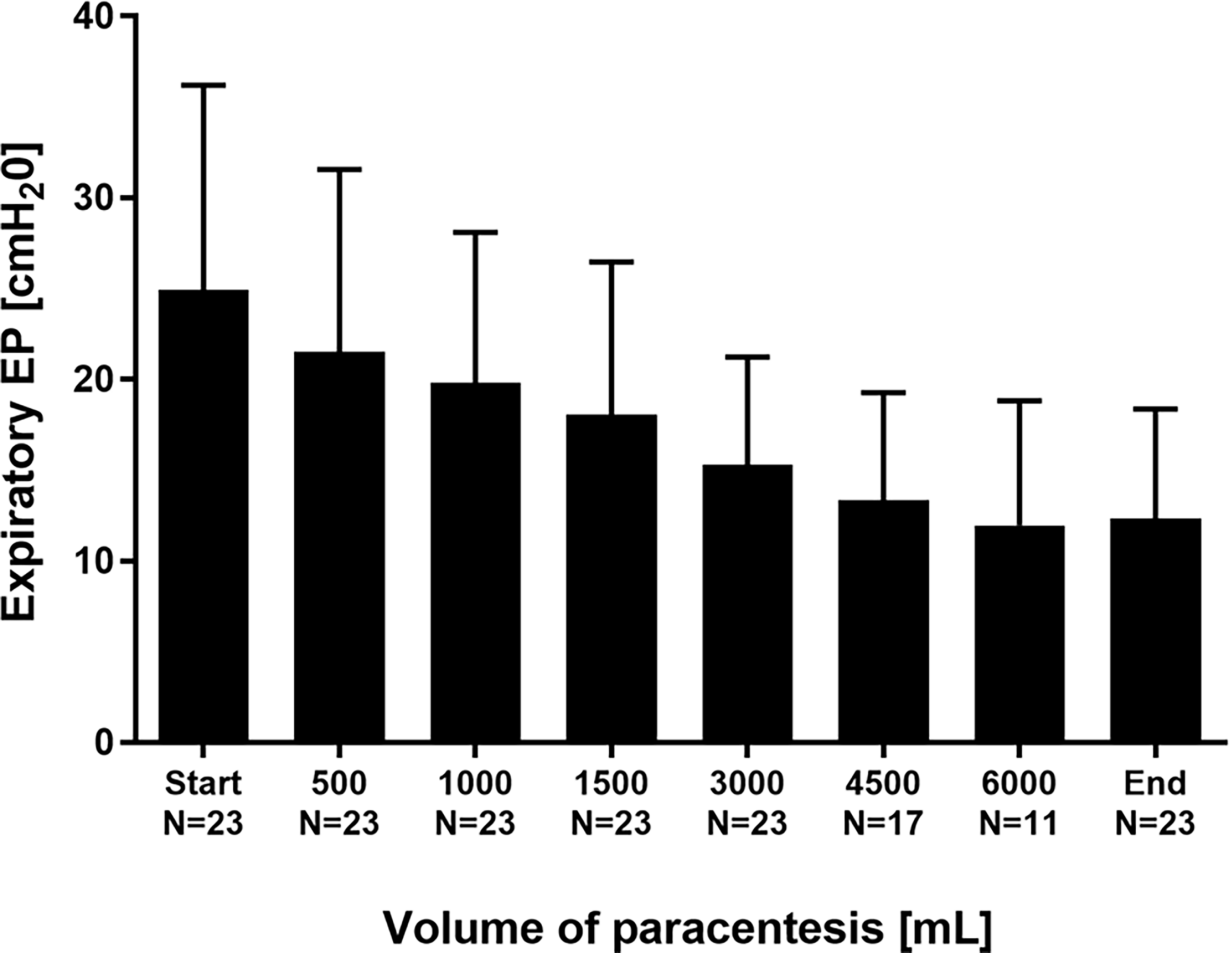
Expiratory esophageal pressure EP in the course of stepwise release of ascites.

Conversely, EP significantly decreased: Inspiratory EP was reduced from 14.1±14.5 to 2.4±8.7 cmH_2_O (p<0.001; Fig 3). Expiratory EP diminished from 24.9±11.3 to 12.4±6.0 cmH_2_O (p<0.001; Fig 4).

The changes of TPP (ΔTPP) and EP (ΔEP) were highest after the release of the *first* 500 mL ascites volume: The course of these changes in pressure levels during stepwise paracentesis from 500 mL to a maximum of 6000 mL ascites is illustrated in Table 2. Due to the different numbers of patients reaching the different volumes of removed ascites no statistical comparisons were performed, and the data shown in Table 2 are predominantly descriptive.

**Table 2:** Changes in TPP (ΔTPP) and EP (ΔEP) during paracentesis compared to the previous measurement.

ΔTPP and ΔEP during stepwise release of ascites (cmH_2_O)					
Removed volume, mL	n	ΔTPP_insp_	ΔTPP_exp_	ΔEP_insp_	ΔEP_exp_
**500 mL**	23	+3.05±2.33	+3.47±3.76	-3.31±3.20	-3.42±3.73
**1000 mL**	23	+2.11±1.49.	+1.32±1.52	-1.88±2.16	-1.71±2.34
**1500 mL**	23	+1.23±2.84	+1.30±1.36	-0.93±2.15	-1.75±2.64
**3000 mL**	23	+2.47±2.78	+3.41±5.03	-2.63±3.60	-2.75±4.43
**4500 mL**	17	+2.66±8.7	+3.26±3.5	-2.50±8.2	-1.95±4.2
**6000 mL**	11	+2.72±0.4	+0.55±1.2	-1.23±0.8	-1.38±1.0

ΔTPP_insp_: Changes in inspiratory transpulmonary pressure

ΔTPP_exp_: Changes in expiratory transpulmonary pressure

ΔEP_insp_: Changes in inspiratory esophageal pressure

ΔEP_exp_: Changes in expiratory esophageal pressure

**Fig 5:**
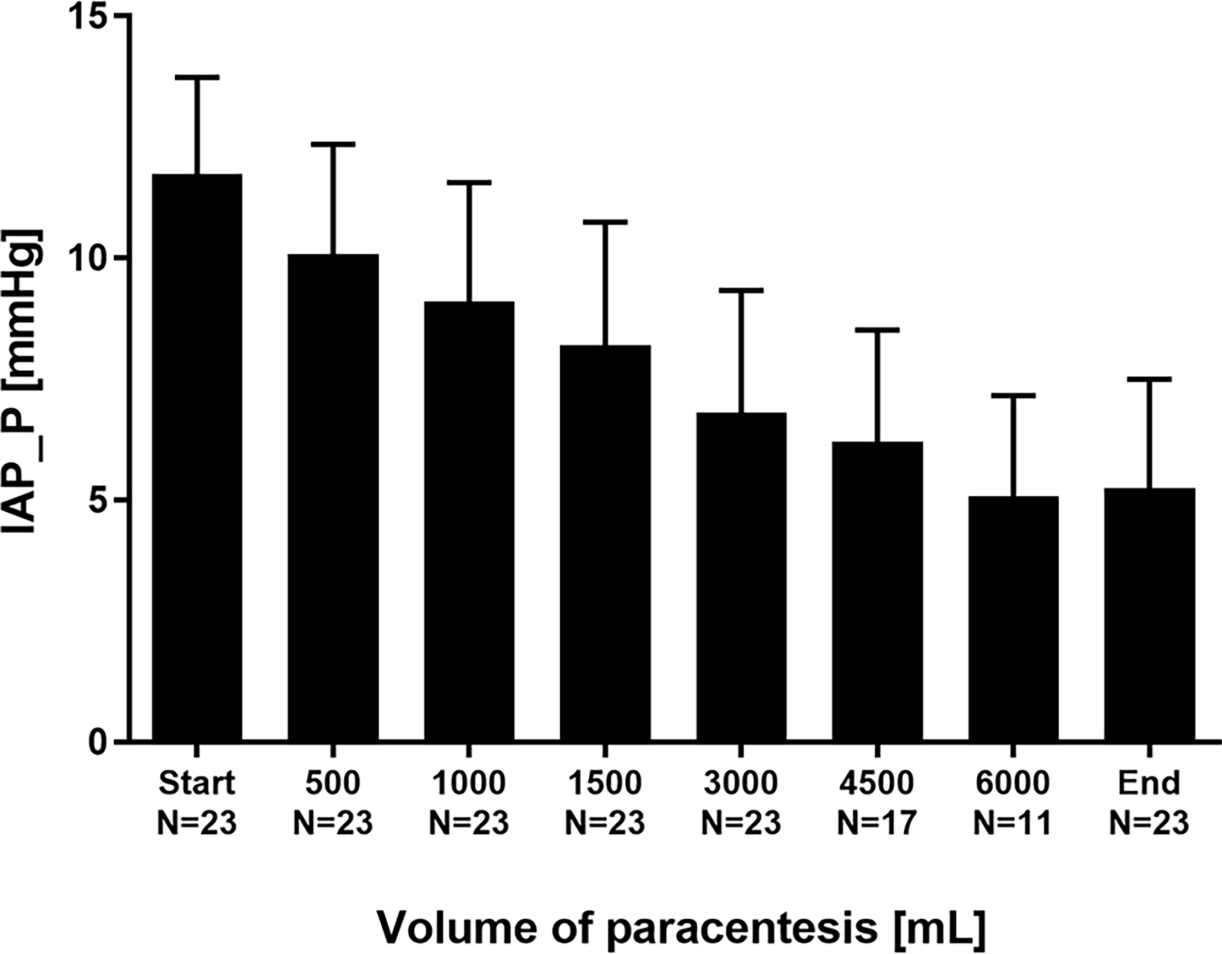
Intraabdominal pressure derived from intra-peritoneal measurement (IAP_P) in the course of stepwise release of ascites.

**Fig 6:**
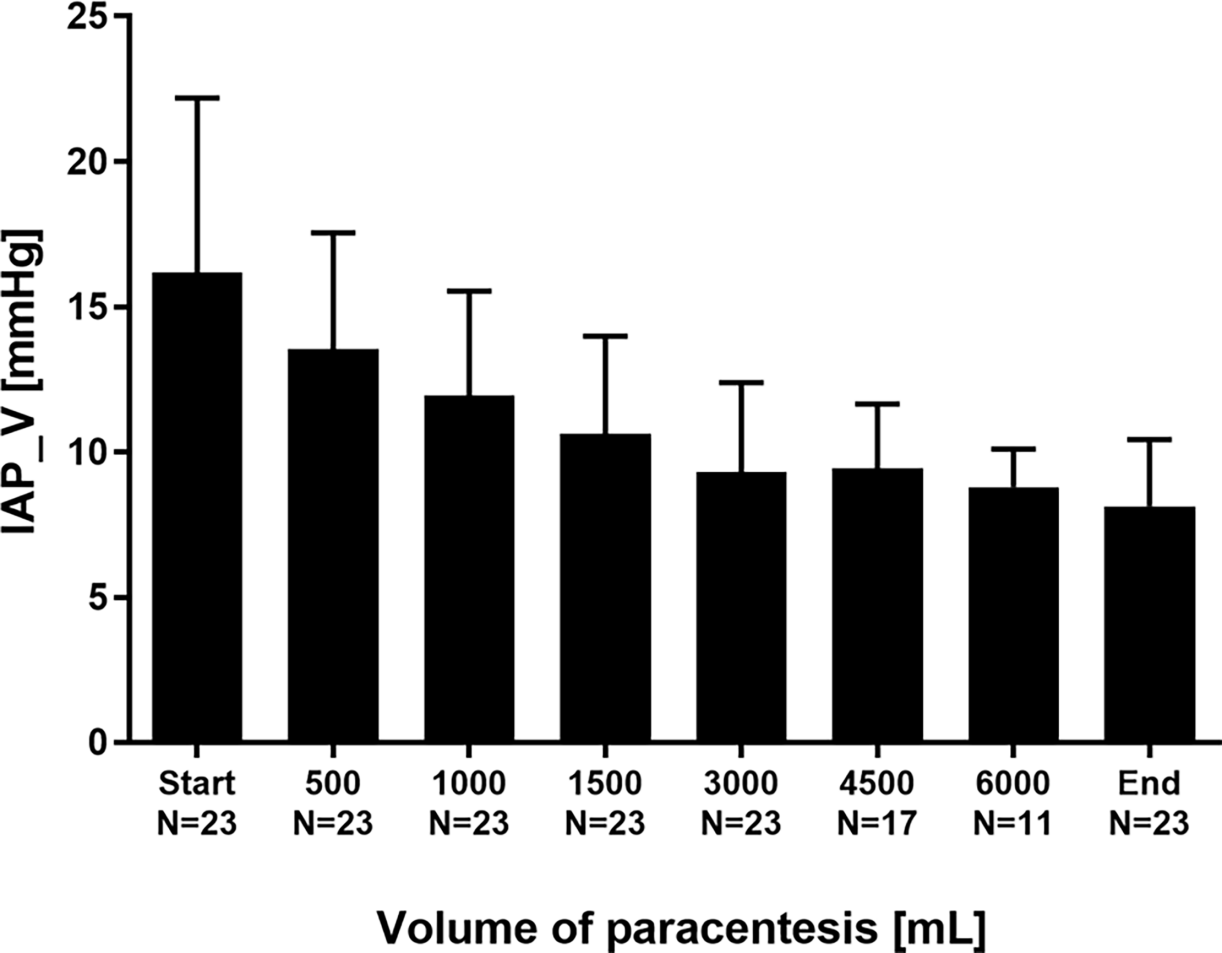
Intraabdominal pressure derived from intra-vesical measurement (IAP_V) in the course of stepwise release of ascites.

Furthermore, LVP resulted in a marked decrease of IAP: At the end of paracentesis, levels of IAP_P had lowered from 11.7±2.0 to 5.2±2.3 mmHg (p<0.001; Fig 5). IAP_V had decreased from 16.2±6.0 to 8.1±2.3 mmHg (p = 0.001; Fig 6). The differences between IAP_P and IAP_V are in line with previous findings of a slight overestimation of intraabdominal pressure by bladder pressure measurement [33]. However, the decreases in IAP_V and IAP_P induced by complete LVP (after LVP vs. before LVP) were not significantly different for both techniques to measure IAP (p = 0.508).

Similarly to changes in TPP and EP, changes in intraabdominal pressures ΔIAP_P and ΔIAP_V were most pronounced for the removal of the *first* 500 mL volume during stepwise paracentesis (Table 3). Due to the different numbers of patients reaching the different volumes of removed ascites no statistical comparisons were performed, and the data shown in Table 3 are predominantly descriptive.

**Table 3:** Changes in intraabdominal pressures (ΔIAP_P and ΔIAP_V) during stepwise paracentesis compared to the previous measurement.

Changes in intraabdominal pressures ΔIAP_P and ΔIAP_V during stepwise paracentesis (mmHg)			
Removed volume, mL	n	ΔIAP_P	Δ IAP_V
**500 mL**	23	-1.65±0.94	-2.64±4.67
**1000 mL**	23	-0.99±0.70	-1.59±1.36
**1500 mL**	23	-0.90±0.53	-1.32±1.15
**3000 mL**	23	-1.39±0.59	-1.32±0.64
**4500 mL**	17	-0.60±0.5	+0.12±1.0
**6000 mL**	11	-1.13±0.9	-0.64±1.3

ΔIAP_P: Changes in intraabdominal pressure (intra-peritoneal measurement)

ΔIAP_V: Changes in intraabdominal pressure (intra-vesical measurement)

### Hemodynamic parameters

LVP reduced central venous pressure CVP from 21.4±9.4 to 15.0±9.7 mmHg (p<0.001). The substantial decrease in CVP by about 30% was in contrast to unchanged values for all hemodynamic parameters assessed by TPTD or pulse contour analysis: Mean arterial pressure MAP (81.0±8.6 vs 78.6±8.4 mmHg, p = 0.193), cardiac Index CI (5.3±1.5 vs 5.6±1.2 L/min/m^2^, p = 0.211), global end-diastolic volume index GEDVI (771.4±103.6 vs 770.9±87.2 mL/m^2^, p = 0.990), extravascular lung water index EVLWI (10.5±4.2 vs 10.8±3.9 mL/kg, p = 0.652) and systemic vascular resistance index SVRI (1013.0±419.8 vs 960.5±369.7 dyn*s*cm^-5*^m^-2^, p = 0.397) did not change significantly (Table 4).

**Table 4:** Hemodynamic parameters assessed by TPTD and CVP-measurement before and after paracentesis.

Hemodynamic parameters assessed by TPTD and CVP-measurement			
	Before paracentesis	After paracentesis	p-value
	Mean ± SD Std. Deviation	Mean ± SD	
**MAP, mmHg**	81.0±8.6	78.6±8.4	0.193
**CI, L/min/m**^**2**^	5.3±1.5	5.6±1.2	0.211
**GEDVI, mL/m**^**2**^	771.4±103.6	770.9±87.2	0.990
**EVLWI, mL/kg**	10.5±4.2	10.8±3.9	0.652
**SVRI,** dyn*s*cm^-5^*m^**-2**^	1013.0±419.8	960.5±369.7	0.397
**CVP, mmHg**	21.4±9.4	15.0±9.7	< 0.001

TPTD: Transpulmonary thermodilution

MAP: Mean arterial pressure

CI: Cardiac index

GEDVI: Global end-diastolic volume index

EVLWI: Extravascular lung water index

SVRI: Systemic vascular resistance index

CVP: Central venous pressure

### Respiratory parameters

All parameters and indicators of respiratory function significantly improved during LVP without changes of the ventilator setting: We registered an improvement of p_a_O_2_/F_i_O_2_ from 208.3±46.8 to 247.7±60.9 mmHg (p<0.001) as well as of Oxygenation index OI from 5.8±3.1 to 4.8±2.0 cmH_2_O/mmHg (p = 0.002).

P_a_O_2_/F_i_O_2_ before all LVP procedures was in line with ARDS according to the Berlin-criteria [34]. After LVP, p_a_O_2_/F_i_O_2_ had substantially improved in four measurements to values above 300mmHg, which is outside the range defining ARDS (see Table 5).

**Table 5:** Classification of p_a_O_2_/F_i_O_2_ according to the Berlin-definition before and after paracentesis.

ARDS according to Berlin definition		
	Before LVP	After LVP
**No**	0/23 (0%)	4/23 (17%)
**Mild**	16/23 (70%)	16/23 (70%)
**Moderate**	7/23 (30%)	3/23 (13%)
**Severe**	0/23 (0%)	0/23 (0%)

Furthermore, LVP induced an increase of tidal volume TV from 452±113 to 510±100 mL (p = 0.008) and of compliance C_dyn_ from 35.1±14.6 to 46.8±15.9 mL/cmH_2_0 (p<0.001). LVP also induced a decrease in p_a_CO_2_ from 51.3±12.2 to 47.3±10.7 (p = 0.046). Based on the high ratio of patients with pressure-supported ventilation in this analysis, we also found a decrease of the respiratory rate from 19.6±7.8 to 17.1±7.3 min^-1^ (p = 0.010). Improvement of respiratory function is outlined in Table 6.

**Table 6:** Respiratory and ventilatory parameters before and after paracentesis.

Respiratory and ventilatory parameters			
	before paracentesis	after paracentesis	p-value
	mean (Std. Deviation) Std. Deviation	mean (Std. Deviation)	
**p**_**a**_**O**_**2**_**/F**_**i**_**O**_**2**_	208.3±46.8	247.7±60.9	< 0.001
**OI, cmH**_**2**_**O/mmHg**	5.8±3.1	4.8±2.0	0.002
**p**_**a**_**CO**_**2**_**, mmHg**	51.2±12.3	47.3.±10.7	0.046
**TV, mL**	452±113	510±100	0.008
**C**_**dyn**_**, mL/cmH**_**2**_**O**	35.1±14.6	46.8±15.9	< 0.001
**Respiratory rate, min**^**-1**^	19.6±7.8	17.1±7.3	0.010

OI: Oxygenation Index

TV: Tidal volume

C_dyn_: Dynamic compliance

p_a_CO_2_: Arterial partial pressure of carbon dioxide

## Discussion

In this study LVP induced an immediate improvement of several parameters of lung function in patients with decompensated liver cirrhosis, tense ascites and high intraabdominal pressure IAP. According to our data this respiratory enhancement is largely referable to changes in transpulmonary pressure TPP.

Optimization of TPP seems to play a key role in critically ill patients with elevated IAP. Adapted levels of TPP improve oxygenation and limit alveolar damage [5, 10]. TPP-guided ventilation has been investigated with promising results in patients with acute lung injury and ARDS [11, 35]. In the present study we used a baseline ventilator setting in line with the current Acute Respiratory Distress Syndrome Network (ARDSNet) recommendations [1, 36]. Under these conditions we noticed overall *negative* expiratory levels for TPP, indicating repeated alveolar collapse and lung atelectasis [37]. LVP provoked a significant increase of TPP with respiratory improvement together with inverse decreases of esophageal pressure EP and IAP.

These findings are supported by previous data underlining the impact of LVP on oxygenation. The benefit of LVP is particularly due to decreases in IAP and consecutive improvement of respiratory mechanics [24, 38, 39]. Additionally, several studies suggest that LVP increases alveolar recruitment and gas exchange in mechanically ventilated patients by enhancing the end-expiratory lung volume as well as C_dyn_ [24, 25]. Our study confirmed this beneficial effect of LVP on respiratory key parameters: The release of ascites resulted in significant increases of p_a_O_2_/F_i_O_2_, C_dyn_ and tidal volume. Moreover, the Oxygenation index OI improved after LVP. OI combines mean airway pressure level with p_a_O_2_/F_i_O_2_ in a single and easily provided parameter. In several studies OI better predicted outcome of ARDS-patients compared to ARDS definitions predominantly based on p_a_O_2_/F_i_O_2_ [40–42]. With regard to the substantial changes of pressure levels during LVP, OI seems to be particularly useful to reflect respiratory function in case of decompensated cirrhosis.

Furthermore, LVP induced a substantial decrease of IAP in our study. Elevations of IAP are common in decompensated liver cirrhosis [6, 15]. Increased IAP is a risk factor for mortality “per se” in critically ill patients. Intraabdominal hypertension decreases abdominal perfusion and frequently results in impairment of renal, cardiovascular and respiratory organ function [16, 17, 43–45]. The evacuation of intraabdominal fluid collections and ascites is one of the few non-surgical options in the management of elevated IAP [46]. In line with this, we found a rapid and significant reduction of markedly elevated baseline IAP with the most pronounced decrease after the *first* 500 mL of evacuated volume. This observation is supported by previous findings that in case of high baseline IAP even a low-volume paracentesis might considerably decrease the pressure level [28].

In contrast to the beneficial effects on TPP, respiratory function and IAP, there were no changes in hemodynamic function after LVP. Previous studies yielded contradictory statements regarding cardiocirculatory parameters: Some of them described mechanisms of paracentesis-induced circulatory dysfunction [47–49], while other data did not find an impairment of the hemodynamic system [50, 51]. Our hemodynamic analyses by transpulmonary thermodilution TPTD and pulse contour analysis revealed steady parameters of hemodynamic function. This favorable result is in tune with a recent trial illustrating that LVP did not restrain the hemodynamic profile assessed by TPTD [25]. Nevertheless, we registered a significant decrease of CVP after paracentesis. Previous studies characterized CVP as an unreliable parameter regarding blood volume, cardiac preload and fluid management, since CVP is considerably depending on extravascular factors such as patient positioning, ventilator setting, intra-thoracic as well as *extra-thoracic* pressure level [32, 52–54]. Therefore, our data with decreases of both IAP and CVP in parallel with paracentesis—again—question CVP as a marker of preload in mechanically ventilated patients with high IAP.

Altogether, the study emphasizes the importance of individualized and optimized ventilator setting in certain patient populations. We used a baseline ventilator setting in accordance with the current Acute Respiratory Distress Syndrome Network (ARDSNet) recommendations [1, 36]. Parameters were adjusted by the treating ICU physician irrespective of the present study. The baseline setting was not changed after the transfer to the AVEA device with initiation of EP and TPP measurement. The overall negative values for expiratory TPP carry the risk of cyclic alveolar collapse. Accordingly, our data show that tense ascites markedly restraines TPP- Consequently, PEEP-setting exclusively based on ARDSNet-recommendations might be unfavorable in case of intraabdominal hypertension. Patients with decompensated cirrhosis or ARDS might benefit from PEEP-setting at a higher level to maintain positive TPP and to counteract IAP. However, any increase of airway and intrathoracic pressure level by higher PEEP necessarily aggravates intraabdominal hypertension [55, 56]. Further increases in IAP might be harmful especially in patients with decompensated cirrhosis [43–45]. By contrast, paracentesis improves both IAP and TPP without the need to further increase the airway pressure level.

Nevertheless, a ventilator setting restricted exclusively to optimized TPP might be difficult to guide in case of high-grade esophageal varices which preclude the measurement of esophageal pressure. Since high-grade varices are frequent in end-stage liver disease, other strategies including continuous or repeated measurements of IAP should be considered in these patients.

### Strengths and limitations

To the best of our knowledge this is the first study analyzing the effects of LVP on TPP, respiratory and ventilatory parameters as well as advanced hemodynamic monitoring data in cirrhotic patients with tense ascites.

Although our results are conclusive with high levels of statistical significance, this is a single centre study with a limited number of patients and datasets.

## Conclusion

In mechanically ventilated patients with decompensated cirrhosis, tense ascites with increased IAP resulted in a substantially decreased TPP despite PEEP-setting according to the ARDSNet.

LVP induced a substantial improvement of TPP, p_a_O_2_/F_i_O_2_, p_a_CO_2_, OI, TV and C_dyn_. CVP decreased in parallel with IAP, while all other hemodynamic parameters remained unchanged by LVP.

## Abbreviations

TPP: transpulmonary pressure
EP: esophageal pressure
IAP: intraabdominal pressure
CVP: central venous pressure
OI: oxygenation Index
C_dyn_: dynamic respiratory system compliance
PEEP: positive end-expiratory pressure
TV: tidal volume
PAW: airway pressure
ARDS: acute respiratory distress syndrome
TPTD: trans-pulmonary thermodilution
p_a_O_2_: arterial partial pressure of oxygen
p_a_CO_2_: arterial partial pressure of carbon dioxide
F_i_O_2_: fraction of inspired oxygen
LVP: large volume paracentesis
IAP_P: IAP determined by intra-peritoneal measurement
IAP_V: IAP determined by intra-vesical measurement
APACHE: acute and physiology chronic health evaluation
SOFA: sequential organ failure assessment
MELD: model of end-stage liver disease
ΔTPP_insp_: changes in inspiratory transpulmonary pressure
ΔTPP_exp_: changes in expiratory transpulmonary pressure
ΔEP_insp_: changes in inspiratory esophageal pressure
ΔEP_exp_: changes in expiratory esophageal pressure
MAP: mean arterial pressure
CI: cardiac index
GEDVI: global end-diastolic volume index
EVLWI: extravascular lung water index
SVRI: systemic vascular resistance index
